# Ovarian Pedicle Local Analgesia in Midline versus Flank Cat Ovariectomy

**DOI:** 10.1002/vms3.70095

**Published:** 2025-02-25

**Authors:** Alice Carbonari, Antonio Di Bello, Edoardo Lillo, Francesco Caprio, Matteo Burgio, Vincenzo Cicirelli, Raffaele Luigi Sciorsci, Annalisa Rizzo

**Affiliations:** ^1^ Department of Veterinary Medicine University of Bari ‘Aldo Moro’ Bari Italy

**Keywords:** cat, flank, lidocaine, midline, ovariectomy

## Abstract

**Objectives:**

The study aimed to assess perioperative pain in cats subjected to ovariectomy, performed with a midline or flank approach, receiving a lidocaine splash on each ovarian pedicle.

**Methods:**

Eighty female cats were divided into two groups: flank group (F), made up of 40 subjects ovariectomised using the flank approach, and the midline group (M), which consisted of 40 subjects using the midline approach. Each group was divided into two subgroups: the local anaesthesia group (L), consisting of 20 cats belonging to group F (LF) and 20 cats belonging to group M (LM), which received a splash of lidocaine (4 mg/kg) diluted in 0.5 mL/kg of saline solution on the ovarian pedicles before their ligation, and the control group (C), consisting of 20 cats belonging to group F (CF) and 20 cats belonging to group M (CM), which received the same splash using saline solution. Heart rate, respiratory rate and systolic blood pressure were monitored to assess intraoperative nociception response. An operator assigned postoperative pain scores following the Glasgow scale revised composite measure pain scale feline.

**Results:**

Intraoperative HR and SBP and postoperative pain scores were higher in the control groups. The number of patients requiring rescue analgesia was higher in the control groups. In the midline approach, HR and SBP increased significantly during exteriorisation of both ovaries, while in the flank approach, this occurred predominantly during exteriorisation of the second ovary.

**Clinical Significance:**

Concurred use of local and systemic analgesia in cats undergoing flank or midline ovariectomy may reduce intraoperative nociception and postoperative pain and reduce the need for rescue analgesia.

## Graphical abstract

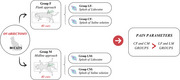

## 1 Introduction

The ovariectomy (OVE) is one of the most common surgeries utilised for the neutering of female cats. Surgery can be performed through either midline or flank laparotomy (McGrath, Hardie, and Davis 2004). In the United Kingdom, 96% of freelance veterinarians prefer the flank approach. In the USA, the midline approach is more widespread (Coe et al. 2006; May 2012). Many studies have attempted to evaluate whether differences exist between these two approaches in terms of duration of surgery, anaesthesia, intraoperative complications, perioperative pain and wound healing. However, in many of them, ovariohysterectomy (OVH) and not OVE were performed, and minimal differences were found among the two approaches (Burrow et al. 2006; Coe et al. 2006; Gauthier et al. 2015; Grint et al. 2006; May 2012; Munif, Safawat, and Hannan 2022; Swaffield, Molloy, and Lipscomb 2020).

OVE is a painful surgical technique where incision of the abdominal wall and manipulation of the ovarian pedicle result in acute nociceptive stimuli. Particular attention should be paid during the traction and ligation of the ovaries because, if analgesia is inadequate, increases in heart rate (HR) and respiratory rate (RR) and pronounced abdominal straining and movement can occur (Bubalo et al. 2008). Pain is defined as ‘a distressing experience associated with actual or potential tissue damage with sensory, emotional, cognitive, and social components’ (Williams and Craig 2016), whereas nociception is ‘the neural process of encoding noxious stimuli’ (St. John Smith 2018). Many techniques have been used to manage OVE nociception and postoperative pain, such as the systemic administration of analgesics in the perioperative period (Warne et al. 2014), peritoneal washing with local anaesthetics (Benito et al. 2016; Grubb et al. 2020), infiltration of local anaesthetics in strategic areas during surgery (Benito et al. 2016; Fudge et al. 2020; Zilberstein, Moens, and Leterrier 2008) and lumbosacral epidural anaesthesia (DeRossi et al. 2016).

The term ‘multimodal balanced anaesthesia’ refers to the concurrent use of different anaesthetic agents and techniques (e.g., premedication, regional and general anaesthesia) to achieve the desired anaesthetic goals, which include analgesia, muscle relaxation, reduction or elimination of autonomic reflexes and maintenance of homeostasis (Brown, Pavone, and Naranjo 2018). The use of more agents and lower doses aims to maximise the desired effect while minimising side effects.

Local anaesthetics bind voltage‐gated sodium channels and block pain signal transmission. The association of local anaesthetics with other systemically administered analgesics (multimodal anaesthesia) induces a more complete analgesia (Berry 2015; Jin and Chung 2001). The guidelines of the American Animal Hospital Association recommend using multimodal analgesia as far as possible for any surgical procedure (Epstein et al. 2015). Using local anaesthetics as part of a multimodal anaesthetic approach helps to abolish or reduce the nociceptive stimulus and decreases the concentration of the anaesthetic required to maintain anaesthesia (Zilberstein, Moens, and Leterrier 2008). Intraperitoneal administration of local anaesthetics reduces the requirement for postoperative analgesia following abdominal surgery (Arden et al. 2013; Buck et al. 2004; De Ol Carapeba et al. 2020).

The aim of this study was to evaluate the efficacy of local ovarian pedicle analgesia, performed by a lidocaine splash, on intraoperative nociception and postoperative pain in cats undergoing OVE via a midline or flank approach. We hypothesised that the association of local and systemic analgesia would improve surgical conditions and reduce postoperative pain in both surgical approaches.

## 2 Materials and Methods

### 2.1 Animals

The study was conducted on 80 crossbred cats. On arrival at the clinic, the cats were placed in individual cages in a room used exclusively for shelter. The day before surgery, all cats were evaluated with a comprehensive physical clinical examination, measurement of packed cell volume and serum total protein and abdominal ultrasonography. Animals with clinical signs of disease, marked aggressiveness, pregnancy, lactation, obesity (body condition score > 7 on a scale of 1 to 9), anaemia and/or cardiac arrhythmias were excluded. Cats younger than 6 months and older than 7 years were also excluded.

### 2.2 Anaesthetic Protocol and Procedures

The cats were fasted for 12 h, and access to water was removed 2 h before surgery. The animals were premedicated with 0.01 mg/kg dexmedetomidine hydrochloride (Dexdomitor; Orion Pharma, Milano MI, Italy, 0.5 mg/mL), 2 mg/kg ketamine hydrochloride (Lobotor, ACME, Cavriago RE, Italy, 100 mg/mL) and 0.02 mg/kg methadone hydrochloride (10 mg/mL, Semfortan, Dechra, Torino TO, Italy) by an intramuscular route (IM). Approximately 5 min later, the cephalic vein was catheterised under aseptic conditions with a 22 or 24 G venous catheter (DeltaVen, Ferrara FE, Italy). Saline solution (0.9% NaCl) was administered at a rate of 3 mL/kg/h intravenously (IV) for the duration of the surgery. Induction of anaesthesia was done using propofol IV (10 mg/mL, Propovet, Zoetis, Rome, Italy) to effect. Once induced, 0.1 mL of lidocaine hydrochloride (Lidocaine 2%, Ecuphar Veterinaria, Barcelona, Spain) was instilled on to the vocal cords, and the animals were intubated with a cuffed endotracheal tube of appropriate size. Anaesthesia was maintained with isoflurane (100% w/w, IsoFlo, Zoetis, Rome, Italy) administered with 100% oxygen using a non‐rebreathing Humphrey A.D.E. in A mode anaesthetic system in a parallel (non‐coaxial) form circuit with a maintenance oxygen flow rate of 100 mL/kg/min via a wall‐mounted Burtons anaesthetic device. Intraoperative monitoring of anaesthesia was performed using a BM5 VET Bionet monitor with side stream capnometer. Spontaneously breathing was maintained in all cats throughout anaesthesia, and no manual ventilation was necessary.

The anaesthetic depth was assessed through clinical evaluations of body movements, jaw tone, eye position, palpebral reflex, heart rate (HR), respiratory rate (RR) and through the evaluation of haemodynamic or respiratory alterations in response to surgical stimuli.

### 2.3 Experimental Design

The animals were randomly divided (using StatView statistical software JMP) into two groups:

1. Flank group (F): consisting of 40 cats undergoing OVE using the flank approach.
2. Midline group (M): consisting of 40 cats undergoing OVE using the ventral midline abdominal approach.

In turn, each group was randomly divided into two subgroups, using the same program:

- Local anaesthesia group (L): consisting of 20 cats belonging to group F (LF) and 20 cats belonging to group M (LM) receiving a topical lidocaine silplash (4 mg/kg), diluted in 0.5 mL/kg of 0.9% saline, on both ovarian pedicles before their ligation.
- Control group (C): consisting of 20 cats belonging to group F (CF) and 20 cats belonging to group M (CM) receiving a saline solution splash on both the ovarian pedicles before their ligation.

The drug solutions were prepared by an operator who was not involved in the management of the animals, and the clinicians involved in anaesthesia and postoperative care were unaware of the administered drugs. All surgical procedures were performed by the same surgeon and by the same operating team.

The flank approach was performed according to the technique described by McGrath, Hardie, and Davis (2004): the cats were placed in left lateral recumbency and a vertical incision (1–1.5 cm) was made between the wing of the ileum and the last rib of the right flank. Skin, external aponeurosis, external and internal oblique abdominal muscles, transverse muscle and peritoneum were incised. Thus, the right ovary was visualised immediately below the incision and was externalised. At this point, the anaesthetist proceeded to splash the ovary with lidocaine, or physiological solution. The surgeon then waited one minute before positioning the haemostatic forceps upstream and downstream of the ovary. A single ligature was placed on the ovarian pedicle, upstream of the haemostat, and another on the ovarian ligament, downstream of the haemostat, using a synthetic absorbable suture (2‐0 Vicryl; Ethicon). Ovary was then removed using a scalpel. To externalise the left ovary, the uterine horn was followed to the bifurcation, and the contralateral horn was exposed with consequent highlighting of the gonad. The left ovary was removed as described for the right. The abdominal wall was closed with absorbable synthetic thread (2‐0 Vicryl; Ethicon), using a simple continuous pattern. Skin closure was performed with a simple interrupted suture pattern using absorbable synthetic thread (2‐0 Vicryl; Ethicon).

The midline approach was performed according to the technique described by Coe et al. (2006): the cats were placed in dorsal recumbency and a horizontal incision was made along the linea alba (1.5–2.0 cm and approximately 1 cm caudal to the umbilicus). The skin, linea alba and peritoneum were incised in the caudocranial direction. The abdominal wall was raised with Allis forceps, and a Snook hook was inserted into the abdominal cavity to hook the uterine horn and externalise it. All procedures for local ovarian pedicle analgesia, OVE, and closure of the operative breach were performed as described for the lateral approach.

Antibiotic therapy with benzylpenicillin (40,000 IU/kg) and dihydrostreptomycin (50 mg/kg) (Repen, FATRO SpA, Ozzano dell'Emilia BO, Italy) was administered IM, and meloxicam 0.3 mg/kg (5 mg/mL, Deflacam, FATRO SpA, Ozzano dell'Emilia BO, Italy) was administered subcutaneously (SC) at the end of the surgery, just prior to anaesthetic recovery. Antibiotic (Amoxicillin trihydrate 11 mg/kg per os—Clamoxyl 200, Zoetis Italia S.r.l., Rome, Italy) and anti‐inflammatory (Meloxicam 0.1 mg/kg per os—Meloxoral, Dechra Veterinary Product S.r.l., Torino, Italy) treatments were continued at home by the owners for 7 and 4 days, respectively.

### 2.4 Nociception and Pain Assessment

Intraoperative nociception and pre‐ and postoperative pain were assessed by the same operator, who was blinded to the use of lidocaine during surgery (Reid, Nolan, and Scott 2018). To ensure pain assessment competence, the operator was trained prior to the study using video footage of cats undergoing OVH. The operator performed a physical examination on all cats before premedication (T0), and an intraoperative nociception assessment was then performed after premedication in the following periods:

· Surgical preparation phase (prior to any surgical procedure) (T1)
· Incision of the abdominal wall (T2)
· Externalisation of the first ovary (T3)
· Manipulation of the first ovarian pedicle (ligatures) 1 min after lidocaine ovarian pedicle analgesia (T4)
· End of manipulation of the first ovarian pedicle after ovary resection (T5)
· Externalisation of the second ovary (T6)
· Manipulation of second ovarian pedicle (ligatures) 1 min after lidocaine local ovarian pedicle analgesia (T7)
· End of manipulation of the second ovarian pedicle after ovary resection (T8)
· End of surgery (affixing the last point on the abdominal skin) (T9)

HR, RR and temperature were detected from T0 to T9. Non‐invasive systolic blood pressure (SBP) using a BM5 VET Bionet monitor and peripheral capillary oxygen saturation (SpO_2_) were detected from T1 to T9. If intra‐operative HR, RR and SBP parameters increased by more than 20% compared to the pre‐incisional (T1) values, fentanyl 0.2 mg/kg (Fentadon, Dechra, Torino TO, Italy 0.05 mg/mL) was administered intravenously (IV) (Skouropoulou et al. 2018). The duration of the surgery was recorded (T2–T9).

Postoperative pain was assessed at 1 (T10), 3 (T11) and 6 h (T12) after extubating, and a score was assigned according to the Glasgow rCMPS‐F scale (Reid et al. 2017). The operator remained blinded to the treatment groups throughout. In the event of Glasgow rCMPS‐F score ≥ 5, buprenorphine 0.015 mg/kg (0.3 mg/mL, Buprefelican, Dechra, Torino TO, Italia) was administered IM (Warne et al. 2014) as rescue analgesia, and the data were recorded.

### 2.5 Statistical Analysis

Compiled forms were entered into a database created with an Excel spreadsheet, and data analysis was performed using Stata MP16 software. Sample size calculation was performed using G*Power for Windows Version 3.1.6 113 (Heinrich Heine Universität Düsseldorf, Germany) (Faul et al. 2007). Continuous variables were described as mean (standard deviation [SD]) and range, and categorical variables as proportions. The skewness and kurtosis tests were used to evaluate the normality of continuous variables; a normalisation model was set up to normalise those that were not normally distributed. The one‐way ANOVA was used to compare continuous variables between groups. The ANOVA for the repeated measures test was used to compare continuous variables between groups and detection time. The Mauchly's test was used to assess the assumption of sphericity of the ANOVA for repeated measures test. For all tests, a two‐sided *p* < 0.05 was considered statistically significant.

## 3 Results

The study sample consisted of 80 female cats: 20 (25.0%) in the LF group, 20 (25.0%) in the CF group, 20 (25.0%) in the CM group and 20 (25.0%) in the LM group. The characteristics of the sample, by group, are described in Table 1. The groups were considered to be homogeneous in terms of weight and age. The mean age of the subjects was 15.6 ± (standard deviation [SD] 3.80) months and the mean body weight was 2.9 ± 0.9 kg (Table 1).

**TABLE 1:** Weight, age and surgery time (mean ± SD) of cats subjected to ovariectomy with midline or flank approach, and with or without local anaesthesia of the ovarian pedicle.

	LF	CF	CM	LM	Total	*p*‐value
Weight	3.2 ± 0.7	2.9 ± 0.8	2.8 ± 0.6	2.8 ± 0.7	2.9 ± 0.9	0.052
Age	17 ± 0.5	17.8 ± 3.1	15.0 ± 2.4	16.6 ± 3.7	15.6 ± 3.8	0.260
Surgery time	20 ± 2	20.8 ± 1	21.9 ± 3	22.1 ± 2.3	22 ± 3.0	0.001

Abbreviations: CF (group control flank); CM (group control midline); LF (group lidocaine flank); LM (group lidocaine midline).

The surgery times differed among the groups (*p* < 0.001); in particular, LM had a higher mean ± SD than other groups (Table 1).

**FIGURE 1:**
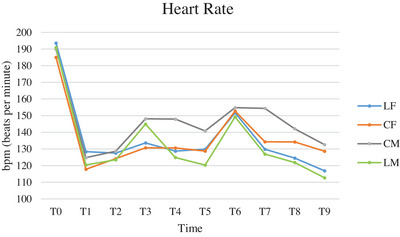
Mean of heart rate (HR), expressed in beats per minute (bpm), of cats subjected to ovariectomy with midline or flank approach, and with or without local anaesthesia of the ovarian pedicle, from the physical exam before premedication (T0) to the end of surgery (T9). CF (group control flank); CM (group control midline); LF (group lidocaine flank); LM (group lidocaine midline); T0 (physical exam before premedication); T1 (surgical preparation phase); T2 (incision of the abdominal wall); T3 (externalisation of the first ovary); T4 (ligature of the first ovarian pedicle 1 min after lidocaine splash); T5 (end of manipulation of the first ovarian pedicle after ovary resection); T6 (externalisation of the second ovary); T7 (ligature of second ovarian pedicle 1min after splash), T8 (end of manipulation of the second ovarian pedicle after ovary resection); T9 (end of surgery).

The trend of HR was similar in all groups (Figure 1): a decrease from T0 to T1 and an increase during exteriorisation of the ovarian pedicles (T3 and T6). ANOVA for repeated measures showed significant differences comparing groups, times and between times and groups (Table 2).

**TABLE 2:** ANOVA analysis for repeated measures of heart rate values (HR) comparing groups, times, and times and groups of cats subjected to ovariectomy with midline or flank approach, and with or without local anaesthesia of the ovarian pedicle.

	Comparison between groups	Comparison between times	Comparison between times and groups
LF vs. CF	0.947	< 0.0001	0.015
LF vs. CM	0.046	< 0.0001	0.001
LF vs. LM	0.376	< 0.0001	0.208
CF vs. CM	0.069	< 0.0001	0.055
CF vs. LM	0.414	< 0.0001	< 0.0001
LM vs. CM	0.015	< 0.0001	< 0.0001

Abbreviations: CF (group control flank); CM (group control midline); LF (group lidocaine flank); LM (group lidocaine midline).

**FIGURE 2:**
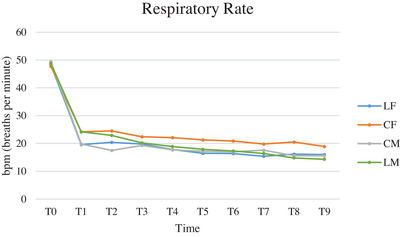
Mean of respiratory rate (RR), expressed in breaths per minute (bpm), of cats subjected to ovariectomy with midline or flank approach, and with or without local anaesthesia of the ovarian pedicle, from the physical exam before premedication (T0) to the end of surgery (T9). CF (group control flank); CM (group control midline); LF (group lidocaine flank); LM (group lidocaine midline); T0 (physical exam before premedication); T1 (surgical preparation phase); T2 (incision of the abdominal wall); T3 (externalisation of the first ovary); T4 (ligature of the first ovarian pedicle 1 min after lidocaine splash); T5 (end of manipulation of the first ovarian pedicle after ovary resection); T6 (externalisation of the second ovary); T7 (ligature of second ovarian pedicle 1 min after splash), T8 (end of manipulation of the second ovarian pedicle after ovary resection); T9 (end of surgery).

The RR trend was similar in all groups, with a tendency to decrease from T0 to T1 and to maintain constant values throughout the surgery (Figure 2). Significant differences between time points and between times and groups are shown in Table 3.

**TABLE 3:** ANOVA analysis for repeated measures of respiratory rate values (RR) comparing groups, times, and times and groups of cats subjected to ovariectomy with midline or flank approach, and with or without local anaesthesia of the ovarian pedicle.

	Comparison between groups	Comparison between times	Comparison between times and groups
LF vs. CF	0.118	< 0.0001	0.520
LF vs. CA	0.992	< 0.0001	0.806
LF vs. LA	0.718	< 0.0001	0.416
CF vs. CA	0.058	< 0.0001	0.009
CF vs. LA	0.294	< 0.0001	0.045
LA vs. CA	0.680	< 0.0001	0.010

Abbreviations: CF (group control flank); CM (group control midline); LF (group lidocaine flank); LM (group lidocaine midline).

**FIGURE 3:**
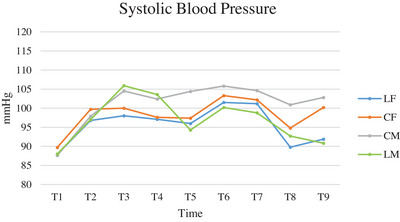
Mean of systemic blood pressure (SBP), expressed in mmHg, of cats subjected to ovariectomy with midline or flank approach, and with or without local anaesthesia of the ovarian pedicle, from the surgical preparation (T1) to the end of surgery (T9). CF (group control flank); CM (group control midline); LF (group lidocaine flank); LM (group lidocaine midline); T1 (surgical preparation phase); T2 (incision of the abdominal wall); T3 (externalisation of the first ovary); T4 (ligature of the first ovarian pedicle 1 min after lidocaine splash); T5 (end of manipulation of the first ovarian pedicle after ovary resection); T6 (externalisation of the second ovary); T7 (ligature of second ovarian pedicle 1 min after splash), T8 (end of manipulation of the second ovarian pedicle after ovary resection); T9 (end of surgery).

SBP values followed a similar trend in CF and LF (Figure 3). CM and LM showed a similar trend from T0 to T4, while from T5, LM had lower values than CM. Significant differences were found between times and between times and groups (Table 4).

**TABLE 4:** ANOVA analysis for repeated measures of systolic blood pressure values (SBP) comparing groups, times, and times and groups of cats subjected to ovariectomy with midline or flank approach, and with or without local anaesthesia of the ovarian pedicle.

	Comparison between groups	Comparison between times	Comparison between times and groups
LF vs. CF	0.458	< 0.0001	0.718
LF vs. CM	0.083	< 0.0001	0.013
LF vs. LM	0.702	< 0.0001	0.030
CF vs. CM	0.310	< 0.0001	0.056
CF vs. LM	0.560	< 0.0001	< 0.0001
LM vs. CM	0.050	< 0.0001	< 0.0001

Abbreviations: CF (group control flank); CM (group control midline); LF (group lidocaine flank); LM (group lidocaine midline).

**FIGURE 4:**
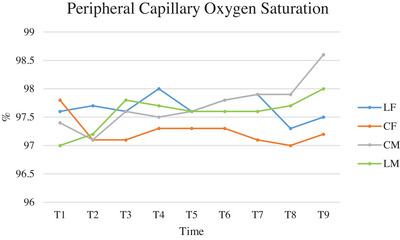
Mean of peripheral capillary oxygen saturation (SpO2), expressed in percentage (%), of cats subjected to ovariectomy with midline or flank approach, and with or without local anaesthesia of the ovarian pedicle, from the surgical preparation (T1) to the end of surgery (T9). CF (group control flank); CM (group control midline); LF (group lidocaine flank); LM (group lidocaine midline); T1 (surgical preparation phase); T2 (incision of the abdominal wall); T3 (externalisation of the first ovary); T4 (ligature of the first ovarian pedicle 1 min after lidocaine splash); T5 (end of manipulation of the first ovarian pedicle after ovary resection); T6 (externalisation of the second ovary); T7 (ligature of second ovarian pedicle 1 min after splash), T8 (end of manipulation of the second ovarian pedicle after ovary resection); T9 (end of surgery).

The values of SpO2 are represented in Figure 4: they were inhomogeneous, but all above 97%. No statistically relevant differences were detected between groups. Significant differences in the comparison between time points and groups were detected in LF versus CM (*p* < 0.004), CF versus CM and LM (*p* < 0.009 and 0.018, respectively) (Table 5).

**TABLE 5:** ANOVA analysis for repeated measures of peripheral capillary oxygen saturation (SpO2) comparing groups, times, and times and groups of cats subjected to ovariectomy with midline or flank approach, and with or without local anaesthesia of the ovarian pedicle.

	Comparison between groups	Comparison between times	Comparison between times and groups
LF vs. CF	0.300	0.151	0.324
LF vs. CM	0.889	0.087	0.004
LF vs. LM	0.826	0.191	0.089
CF vs. CM	0.266	0.096	0.009
CF vs. LM	0.493	0.754	0.018
LM vs. CM	0.749	0.001	0.734

Abbreviations: CF (group control flank); CM (group control midline); LF (group lidocaine flank); LM (group lidocaine midline).

**FIGURE 5:**
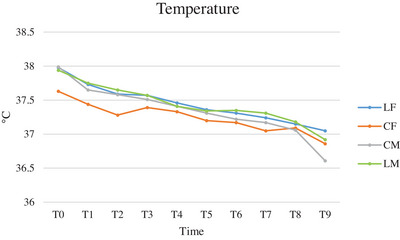
Mean of temperature (T), expressed in degree Celsius (°C), of cats subjected to ovariectomy with midline or flank approach, and with or without local anaesthesia of the ovarian pedicle, from the physical exam before premedication (T0) to the end of surgery (T9). CF (group control flank); CM (group control midline); LF (group lidocaine flank); LM (group lidocaine midline); T0 (physical exam before premedication); T1 (surgical preparation phase); T2 (incision of the abdominal wall); T3 (externalisation of the first ovary); T4 (ligature of the first ovarian pedicle 1 min after lidocaine splash); T5 (end of manipulation of the first ovarian pedicle after ovary resection); T6 (externalisation of the second ovary); T7 (ligature of second ovarian pedicle 1 min after splash), T8 (end of manipulation of the second ovarian pedicle after ovary resection); T9 (end of surgery).

In all groups, rectal temperature tended to decrease from T0 to T9 (Figure 5). While there was no difference between groups, a significant difference was present between the time points (T0‐T12) in all the groups (Table 6).

**TABLE 6:** ANOVA analysis for repeated measures of temperature (T) comparing groups, times, and times and groups of cats subjected to ovariectomy with midline or flank approach, and with or without local anaesthesia of the ovarian pedicle.

	Comparison between groups	Comparison between times	Comparison between times and groups
LF vs. CF	0.237	< 0.0001	0.105
LF vs. CM	0.603	< 0.0001	0.001
LF vs. LM	0.992	< 0.0001	0.737
CF vs. CM	0.559	< 0.0001	< 0.0001
CF vs. LM	0.306	< 0.0001	0.018
LM vs. CM	0.653	< 0.0001	0.070

Abbreviations: CF (group control flank); CM (group control midline); LF (group lidocaine flank); LM (group lidocaine midline).

During postoperative pain assessment, the number of animals requiring rescue analgesia was higher in the control groups for both surgical approaches: two LF cats, four CF cats, two LM cats and four CM cats.

**FIGURE 6:**
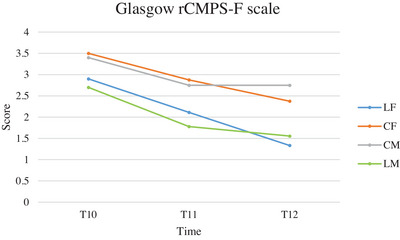
Mean of Glasgow rCMPS‐F scale scores of cats subjected to ovariectomy with midline or flank approach, and with or without local anaesthesia of the ovarian pedicle, from 1 (T10), 3 (T11) and 6 (T12) hours after extubating. CF (group control flank); CM (group control midline); LF (group lidocaine flank); LM (group lidocaine midline); T10 (1 h after extubating); T11 (3 h after extubating); T12 (6 h after extubating).

The Glasgow rCMPS‐F scores are reported in Figure 6. Significant differences comparing groups, times and between times and groups are reported in Table 7.

**TABLE 7:** ANOVA analysis for repeated measures of the Glasgow rCMPS‐F scale scores, comparing groups, times, and times and groups of cats subjected to ovariectomy with midline or flank approach, and with or without local anaesthesia of the ovarian pedicle.

	Comparison between groups	Comparison between times	Comparison between times and groups
LF vs. CF	0.002	< 0.0001	0.048
LF vs. CM	0.001	< 0.001	< 0.001
LF vs. LM	0.874	< 0.001	0.059
CF vs. CM	0.607	0.007	0.007
CF vs. LM	0.013	< 0.0001	0.235
LM vs. CM	0.007	0.018	0.018

Abbreviations: CF (group control flank); CM (group control midline); LF (group lidocaine flank); LM (group lidocaine midline).

## 4 Discussion

The aim of this study was to evaluate the intra‐ and postoperative efficacy of a local ovarian pedicle analgesia in cats undergoing bilateral OVE performed using a flank or midline approach.

As expected based on previous studies (McGrath, Hardie, and Davis 2004), surgery times for the flank approach were shorter than those using the midline approach. A limitation of this study could be represented by the experience of the surgeon with the flank approach, as this could lengthen the surgery times.

HR, RR and SBP values remained within clinically acceptable parameters throughout intraoperative nociception assessment; thus, no intraoperative analgesic supplementation was required.

HR and SBP are standard parameters used for the evaluation of acute intraoperative nociception (Ruíz‐López, Domínguez, and del Granados 2020; Cicirelli, Lacalandra, and Aiudi 2022). The higher HR values in the control groups confirm the preventive effect of lidocaine on nociception (Zilberstein, Moens, and Leterrier 2008). The increases in HR (less than 20% of pre‐incisional HR) observed during externalisation of the first and second ovary (T3 and T6) in the midline group and during the second ovary exteriorisation (T6) in the flank group could be explained by the different position of the ovaries in the two approaches. In the flank approach, the first ovary is located immediately under the incision site and the pedicle is not strained during its ligation. (Swaffield, Molloy, and Lipscomb 2020) Since the second ovary is deeper than the surgical breach, greater traction is required, resulting in a greater nociceptive stimulus.

The tendency for RR to decrease from T0 to T1 is assumed to be a consequence of the anaesthesia. In all groups, RR then followed the trend expected of an optimal analgesic protocol, remaining constant throughout the duration of surgery.

In the SBP comparisons between times and groups, LF cats had lower SBP values during manipulation of the first ovary compared to LM and CM cats. During exteriorisation and manipulation of the second ovary, SBP was higher in LF than LM and lower in LM than CM. As for the HR values, this trend could be explained by the position of the ovaries and by the analgesic effect of lidocaine. LM cats had higher SBP than the control groups during exteriorisation and manipulation of the first ovary, but these values tended to fall below those of the control groups from T5 to the end of surgery. This finding could also be attributable to the analgesic effect of lidocaine and the different positions of the ovaries affecting the manipulations necessary for their externalisation. The lateral approach allows nociception to be limited to a shorter time span than the median approach, although both approaches generate a similar level of nociception at the end of the intervention (Swaffield, Molloy, and Lipscomb 2020).

The number of animals requiring postoperative rescue analgesia in the control groups was double that of the lidocaine groups, implying that the intraoperative use of a local ovarian pedicle analgesia reduces the development of postoperative pain (Zilberstein, Moens, and Leterrier 2008).

Similarly, in the postoperative pain assessment, the control groups (especially CM) had the highest score on the Glasgow rCMPS‐F scale (score 3). In the comparison between times and groups, the CM had higher values at T10 and T12 than the CF, suggesting that the flank approach is better tolerated by the cats (Gauthier et al. 2015). In the lidocaine groups, the number of subjects with the highest pain score was low (10%), and many were assessed as being pain‐free (score 0). The results suggest that the use of intraoperative ovarian pedicle analgesia reduces postoperative pain, and this effect is particularly evident 6 h postextubation (T12).

## 5 Conclusions

A midline surgical approach appears to trigger nociception during exteriorisation of both ovaries, while, using the flank approach, nociception is triggered predominantly during exteriorisation of the second ovary. Local ovarian pedicle analgesia reduces intraoperative nociception and postoperative pain and reduces the need for rescue analgesia. As such, it plays a pivotal role in a multimodal anaesthesia approach for OVE in cats.

## Author Contributions

**Alice Carbonari**: data curation. **Antonio Di Bello**: methodology, writing–original draft, writing–review and editing. **Edoardo Lillo**: investigation, writing–original draft. **Francesco Caprio**: conceptualisation. **Matteo Burgio**: data curation. **Vincenzo Cicirelli**: conceptualisation, writing–review and editing. **Raffaele Luigi Sciorsci**: project administration. **Annalisa Rizzo**: supervision.

## Ethics Statement

All procedures were conducted in compliance with the institutional guidelines on the welfare and use of live animals with the informed consent of the owner and the approval of the ethics committee of the University of Bari ‘Aldo Moro’ (protocol no. 10/2021). The study was conducted at the Department of Veterinary Medicine of the University of Bari ‘Aldo Moro’.

## Conflicts of Interest

The authors declare no conflicts of interest.

### Peer Review

The peer review history for this article is available at https://publons.com/publon/10.1002/vms3.70095.

## Data Availability

The data presented in this study are available on request from the corresponding author.
